# Inhibition of protein kinase II (CK2) prevents induced signal transducer and activator of transcription (STAT) 1/3 and constitutive STAT3 activation

**DOI:** 10.18632/oncotarget.1852

**Published:** 2014-03-23

**Authors:** Samadhi Aparicio-Siegmund, Jan Sommer, Niloufar Monhasery, Ralf Schwanbeck, Eric Keil, David Finkenstädt, Klaus Pfeffer, Stefan Rose-John, Jürgen Scheller, Christoph Garbers

**Affiliations:** ^1^ Institute of Biochemistry and Molecular Biology II, Medical Faculty, Heinrich-Heine University, Düsseldorf, Germany;; ^2^ Institute of Biochemistry, Christian-Albrechts-University, Kiel, Germany;; ^3^ Institute of Medical Microbiology and Hospital Hygiene, Heinrich-Heine University, Düsseldorf, Germany; ^4^ Present address: Institute of Biochemistry, Christian-Albrechts-University, Kiel, Germany

**Keywords:** STAT3, cytokines, tumor, oncogene, signal transduction

## Abstract

The Janus kinase / signal transducer and activator of transcription (Jak/STAT) pathway can be activated by many different cytokines, among them all members of the Interleukin (IL-)6 family. Dysregulation of this pathway, resulting in its constitutive activation, is associated with chronic inflammation and cancer development. In the present study, we show that activity of protein kinase II (CK2), a ubiquitously expressed serine/threonine kinase, is needed for induced activation of STAT1 and STAT3 by IL-6 classic and trans-signaling, IL-11, IL-27, oncostatin M (OSM), leukemia inhibitory factor (LIF) and cardiotrophin-1 (CT-1). Inhibition of CK2 efficiently prevented STAT phosphorylation and inhibited cytokine-dependent cell proliferation in a Jak1-dependent manner. Conversely, forced activation of CK2 alone was not sufficient to induce activation of the Jak/STAT signaling pathway. Inhibition of CK2 in turn inhibited Jak1-dependent STAT activation by oncogenic gp130 mutations. Furthermore, CK2 inhibition diminished the Jak1- and Src kinase-dependent phosphorylation of a constitutively active STAT3 mutant recently described in human large granular lymphocytic leukemia. In conclusion, we characterize CK2 as an essential component of the Jak/STAT pathway. Pharmacologic inhibition of this kinase is therefore a promising strategy to treat human inflammatory diseases and malignancies associated with constitutive activation of the Jak/STAT pathway.

## INTRODUCTION

Activation of the Janus kinase / signal transducer and activator of transcription (Jak/STAT) pathway is induced by numerous biological factors, among them cytokines, interferons and growth factors [1]. Jak/STAT signaling is important for development, growth control and cellular homeostasis [2]. Overshooting activation is prevented through several intracellular negative feedback mechanisms [3]. Nevertheless, there are several mutations described in key proteins of this signaling pathway that confer constitutive activation of Jak/STAT signaling, which lead to chronic inflammatory diseases and cancer development [4, 5].

The pleiotropic cytokines of the Interleukin 6 (IL-6) family are critically involved in numerous physiologic as well as pathophysiologic conditions, thereby facilitating pro- as well as anti-inflammatory properties [3, 6]. The family consists of its members IL-6, IL-11, IL-27, IL-30, IL-31, leukemia inhibitory factor (LIF), oncostatin M (OSM), ciliary neurotrophic factor (CNTF), cardiotrophin-1 (CT-1) and cardiotrophin-like cytokine (CLC) [3]. All cytokines bind to specific receptor complexes on their target cells. With the exception of IL-31, which signals through a heterodimer of GPL and OSMR, all IL-6 type cytokines engage at least one molecule of the ubiquitously expressed β-receptor glycoprotein 130 (gp130). Gp130 can build up a homodimer (induced by IL-6, IL-11 or IL-30 [7, 8]), or form heterodimers with WSX-1 (induced by IL-27 [9]), LIFR (induced by CT-1, OSM or LIF) or OSMR (induced by OSM). Thus, gp130 possesses a remarkable cytokine plasticity, which enables a single cytokine receptor to engage signaling by different ligands via multiple defined interaction sites [3].

IL-6 type cytokine signaling complex formation, irrespective of the β-receptor composition that is used by the individual cytokines, activates the Jak/STAT pathway through Jak1, Jak2 and Tyk2 kinases. Depending on stimulus and cell type, the phosphatidyl-inositol-3-kinase (PI3K)-cascade and the mitogen activated protein kinase (MAPK)-cascade are also activated. Janus kinases are constitutively associated with gp130 [10, 11], and their activation leads to tyrosine phosphorylation of specific motifs within the intracellular domain of gp130 (for a detailed review see [12]). These phosphorylated motifs serve as docking sites for STAT proteins, which are in turn phosphorylated by JAKs and translocate into the nucleus after homo- and/or heterodimerization. IL-6 activates gene transcription mainly via STAT1 and STAT3 proteins [13]. Whether all cytokines activate the same pattern of STAT proteins and whether there are differences concerning strength and duration of this activation is largely unknown and furthermore might be cell-type specific.

Several intracellular proteins have been described that are needed for efficient termination of cytokine-induced signaling, including members of the suppressor of cytokine signaling (SOCS) family or phosphatases such as SHP2 [3]. Interestingly, catalytic activity of protein kinase II (formerly termed casein kinase II (CK2)) was recently shown to be a prerequisite for activation of the Jak/STAT signaling pathway by OSM [14]. CK2 consists of two catalytic α- and two regulatory β-subunits. CK2 is ubiquitously expressed and phosphorylates more than 300 substrates on serine and threonine residues [15, 16]. Genetic knockout of both CK2α and CK2β results in mouse embryonic lethality [17-19], underlining the pivotal role of CK2 in diverse cellular processes such as proliferation, apoptosis and cell division. Furthermore, dysregulated activity of CK2 has been implicated in the development and progression of several hematopoietic tumors, including chronic lymphatic leukemia, multiple myeloma and chronic myeloproliferative neoplasms [20]. Overall, overexpression and increased activity of CK2 has been implicated in the pathogenesis of several types of tumors [21]. Therefore, specific inhibition of CK2 in tumor cells might be an appropriate therapeutic option [22]. CX-4945 (developed by Cylene Pharmaceuticals, San Diego, CA, USA), which blocks CK2α in an ATP-competitive manner, has recently successfully passed phase I clinical trials [23], and the cell-permeable peptide inhibitor CIGB-300 has been shown to be efficient *in vitro* and *in vivo* [24].

In this study, we show that CK2 activity is needed for initiation of Jak/STAT signaling by IL-6 classic and trans-signaling, IL-11, IL-27, oncostatin M (OSM), leukemia inhibitory factor (LIF), and cardiotrophin-1 (CT-1), and that interfering with this signaling pathway critically depends on Jak1. Blockade of CK2 also inhibited a constitutive gp130 variant found in human inflammatory hepatocellular adenomas as well as a constitutive active STAT3 mutant recently described in human large granular lymphocytic leukemia. In summary, we characterize CK2 as an essential component of the Jak/STAT signaling pathway.

## RESULTS

### Activity of protein kinase II (CK2) is necessary for STAT-activation by IL-6 family cytokines

**Fig 1:**
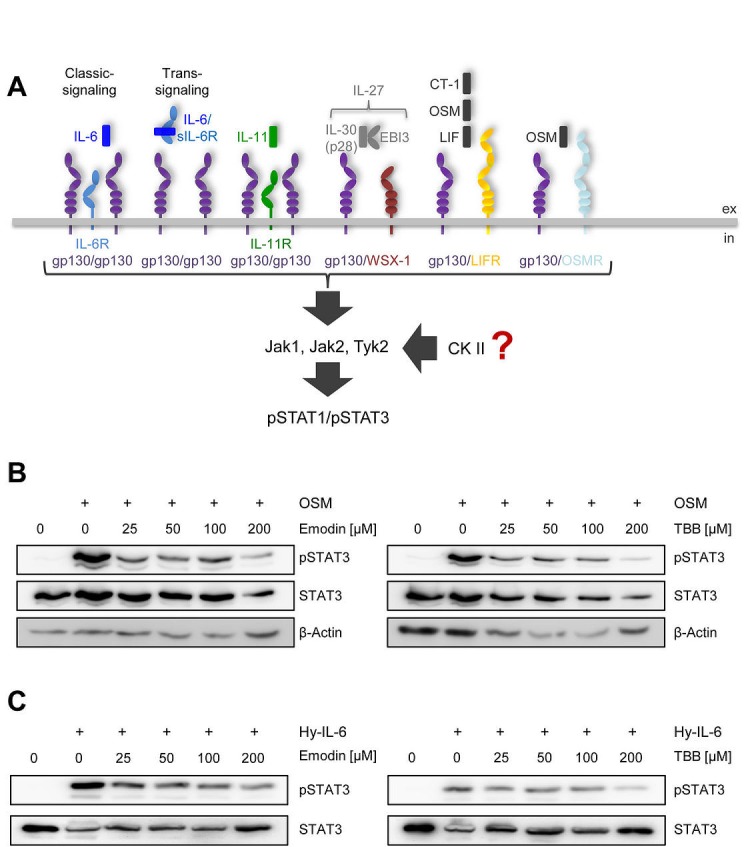
CK2 is involved in STAT3 activation by OSM and Hyper-IL-6 (A) Schematic overview of the members of the IL-6 cytokine family and their receptors investigated in this study. IL-6 can activate a homodimer of glycoprotein 130 (gp130) either via the membrane-bound IL-6R (classic signaling) or via the soluble IL-6R (trans-signaling), whereas IL-11 acts only via a membrane-bound IL-11R. IL-27 (p28/IL-30 and EBI3) engages a gp130/WSX-1 heterodimer. The three members CT-1, OSM and LIF share a heterodimer of gp130/LIFR as signal transduction complex, while OSM can in addition also activate gp130 in combination with OSMR. IL-6 family cytokines activate the three kinases Jak1, Jak2 and Tyk2, which in turn phosphorylate STAT1 and STAT3. The influence of CK2 on this signaling pathway is investigated in the current study. (B) HepG2 cells were treated with different concentrations of the two CK2-inhibitors Emodin and TBB for 90 min. Cells were afterwards stimulated with 10 ng/ml OSM for 15 min. Phosphorylation of STAT3 was assessed by Western blotting. (C) HepG2 cells were treated as described under panel B, but were stimulated with 10 ng/ml Hyper-IL-6. Phosphorylation of STAT3 was assessed by Western blotting. One representative experiment of two performed is shown.

Activation of the Jak/STAT signaling pathway is a hallmark of all IL-6 family cytokines (Figure 1A). Among the seven members of the STAT family, predominantly STAT1 and STAT3 are phosphorylated in response to cytokine-receptor activation [3]. Although this pathway is known for more than 20 years [1], protein kinase II (CK2, casein kinase II) has only recently been shown to be needed for oncostatin-M (OSM)-meditated STAT activation [14]. To verify this, we incubated human liver carcinoma cells (HepG2) with increasing amounts of either Emodin or 4,5,6,7-Tetrabromo-2-azabenzimidazole (TBB), two specific CK2 inhibitors. After 90 min, we stimulated the cells with 10 ng/ml OSM for 15 min und determined STAT3 activation via Western blotting. As shown in Figure 1B, both inhibitors prevented STAT3 phosphorylation in a concentration-dependent manner.

Next, we asked if the CK2-dependent phosphorylation of STAT3 is restricted to OSM, which signals through either gp130/LIFR or gp130/OSMR heterodimers. To address this, we stimulated HepG2 cells with Hyper-IL-6. Hyper-IL-6 is a fusion protein of IL-6 and the soluble IL-6R, which mimics IL-6 trans-signaling and activates a gp130 homodimer [25]. Both inhibitors led to a dose-dependent reduction of Hyper-IL-6-induced STAT3 phosphorylation (Figure 1C). These data suggest a requirement of CK2 for other members of the IL-6 family of cytokines.

**Fig 2:**
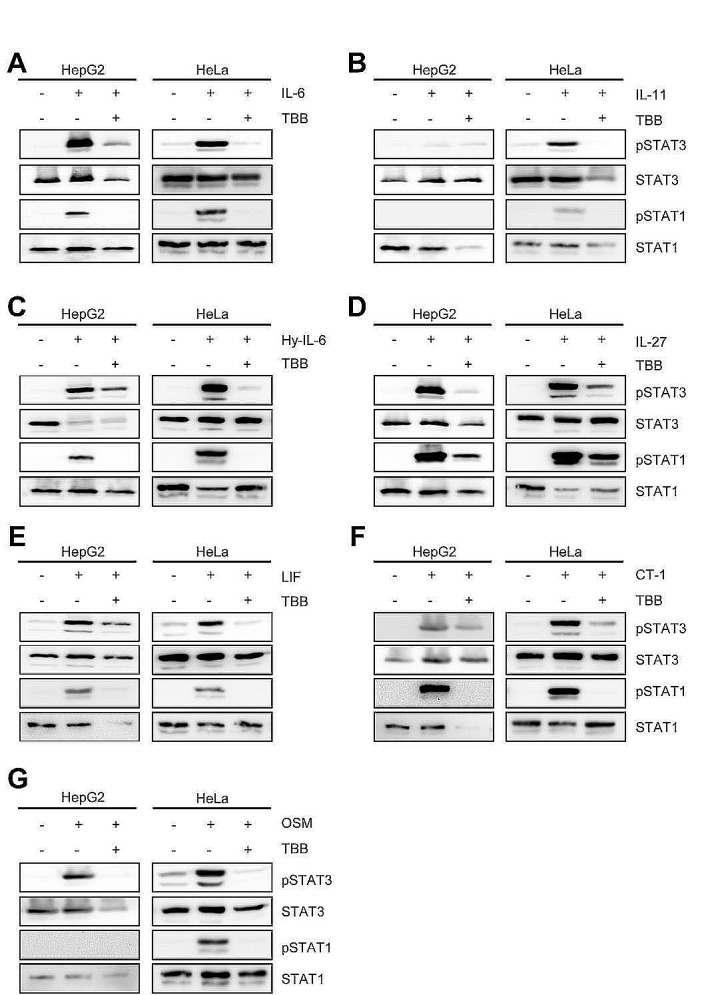
Inhibition of CK2 blocks IL-6 family induced STAT signaling Serum-starved HepG2 or HeLa cells were stimulated with the indicated cytokines for 15 min: (A) HepG2: 10 ng/ml IL-6; HeLa: 20 ng/ml IL-6, (B) HepG2: 10 ng/ml IL-11; HeLa: 20 ng/ml IL-11, (C) 10 ng/ml Hyper-IL-6, (D) 10 ng/ml IL-27, (E) 10 ng/ml LIF, (F) 10 ng/ml CT-1 and (G) 10 ng/ml OSM. The cells were pre-incubated with the CK2 inhibitor TBB (100 μM) for 90 min where indicated. Phosphorylation of STAT1 and STAT3 was assessed by Western blotting, and STAT1/STAT3 served as internal loading control, respectively. One representative Western blot from at least three independent experiments is shown.

Therefore, we decided to systematically address whether CK2 activity is required for the initiation of Jak/STAT signaling by IL-6 family cytokines. IL-6-type cytokines activate distinct ß-receptor complexes that are homo- or heterodimers of the trans-membrane receptors gp130, WSX-1, LIFR and OSMR (Figure 1A) and mainly induce STAT1 and STAT3 phosphorylation (Figure 1A). First, we investigated signaling of IL-6, IL-11 and Hyper-IL-6, which all activate a gp130 homodimer (Figure 1A). Stimulation of HepG2 cells with IL-6 resulted in phosphorylation of STAT1 and STAT3 (Figure 2A). Since emodin and TBB were equally efficient to suppress STAT3 activation (Fig. 1B and C), we conducted the following experiments with 100 μM TBB. Pre-incubation of the cells with this inhibitor almost completely blocked STAT1/STAT3 phosphorylation (Figure 2A), and the same was seen when HeLa cells were stimulated with IL-6 (Figure 2A). HepG2 cells express only little IL-11R and did not respond robustly towards stimulation with 10 ng/ml IL-11 (Figure 2B). HeLa cells, however, have been shown to endogenously express IL-11R [26], and stimulation with IL-11 resulted in STAT1 and STAT3 phosphorylation, which was prevented by CK2 inhibition (Figure 2B). Finally, phosphorylation of STAT1 and STAT3 induced by Hyper-IL-6 was also largely absent in both HepG2 and HeLa cells when CK2 was blocked by TBB (Figure 2C).

Interleukin-27 is the only known cytokine that signals through the β-receptor combination gp130/WSX-1 (Figure 1A) and has been shown to predominantly activate STAT1 [9, 27]. Therefore, we tested whether also Jak/STAT activation by this β-receptor composition relies on CK2 activity. As shown in Figure 2D, IL-27 treatment of HepG2 and HeLa cells led to a strong increase in pSTAT1 and pSTAT3, which was again largely absent when CK2 was blocked by TBB. For the last β-receptor complex, gp130/LIFR, we tested the three known ligands LIF, CT-1 and OSM (Figure 1A). As shown in Figures 2E-G, all three cytokines induced STAT1 and STAT3 phosphorylation in both HepG2 and HeLa cells, and STAT activation was again prevented when cells were pre-treated with TBB.

We concluded from these experiments that CK2 is generally needed for activation of the Jak/STAT pathway by IL-6 type cytokines, and that this is independent from the individual composition of the β-receptor complex.

### Blockade of CK2 inhibits activation of MAPK and PI3K signaling pathways

**Fig 3:**
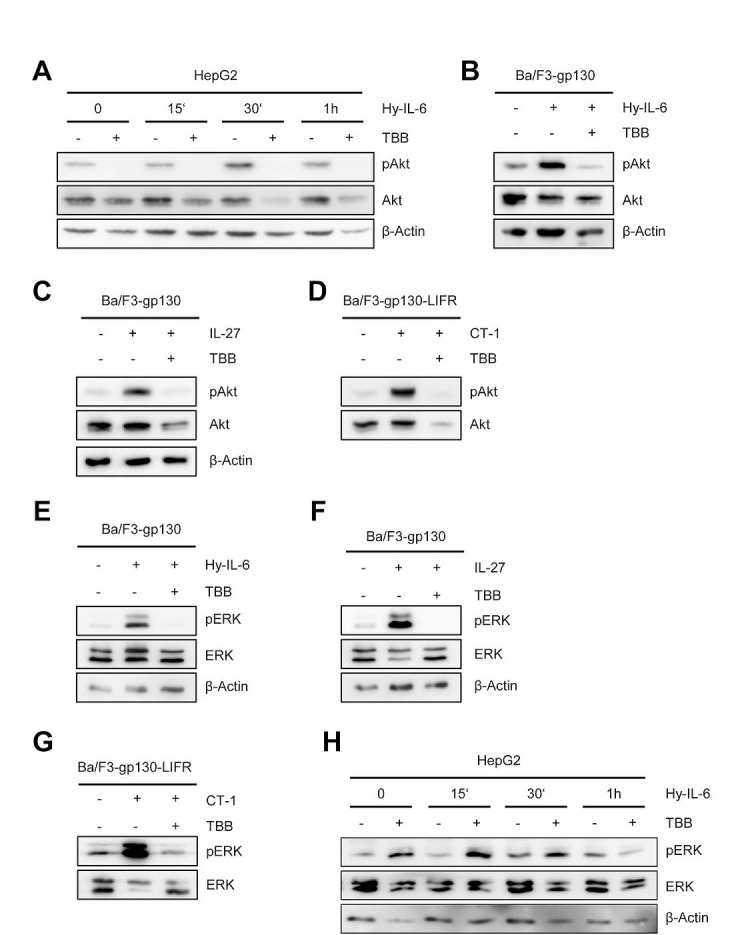
Inhibition of CK2 blocks ERK and Akt signaling (A) HepG2 cells were stimulated with 10 ng/ml Hyper-IL-6, and cells were harvested after 0, 15, 30 and 60min. Cells were either pre-treated with 100 μM TBB for 90 min or with DMSO as control. Phosphorylation and expression level of Akt were assessed by Western blotting, and β-Actin served as internal loading control. (B, C) Ba/F3-gp130 cells were stimulated with 10 ng/ml Hyper-IL-6 or 10 ng/ml IL-27. Cells were either pre-treated with 100 μM TBB for 90 min or with DMSO as control. Phosphorylation of Akt was assessed by Western blotting. (D) The experiment was performed as described under panel C, but Ba/F3-gp130-LIFR cells were stimulated with 10 ng/ml CT-1. (E-G) The experiments were performed as described under panel B-D. Phosphorylation and expression level of ERK were assessed by Western blotting, and β-actin served as internal loading control. (H) HepG2 cells were stimulated with 10 ng/ml Hyper-IL-6, and cells were harvested after 0, 15, 30 and 60 min. Cells were either pre-treated with 100 μM TBB for 90 min or with DMSO as control. Phosphorylation and expression levels of ERK were assessed by Western blotting, and β-actin served as internal loading control. One representative Western blot from at least three independent experiments is shown.

Besides Jak/STAT, IL-6-type cytokines activate the phosphatidyl-inositol-3-kinase (PI3K)-cascade and the mitogen activated protein kinase (MAPK)-cascade. CK2 activity has been shown to be required for efficient initiation of the ERK-MAPK-cascade through its interaction with the molecular scaffold kinase suppressor of ras (KSR) [28]. Furthermore, CK2 is able to phosphorylate Akt, and this phosphorylation is needed for activation of the PI3K cascade [29]. Based on this, we asked whether IL-6 type cytokine-induced activation of ERK and Akt is blocked through inhibition of CK2. As shown in Figure 3A, stimulation of HepG2 cells with Hyper-IL-6 induced a time-dependent phosphorylation of Akt, which peaked 30 min after stimulation. Cells pre-treated with TBB did not show any increase in Akt phosphorylation. Interestingly, the basal phosphorylation of Akt observed in HepG2 cells without any stimulus was absent after treatment of the cells with TBB for 90 min. Furthermore, the protein level of Akt declined over time in TBB-treated cells, which is in line with previous reports [23, 29].

We verified these observations in a second cell line. Ba/F3 cells are murine pre B cells, which only grow in the presence of IL-3. WSX-1 is the only IL-6 family cytokine receptor that is endogenously expressed in these cells, but stable transduction with other cytokine receptors renders them responsive to the respective cytokines [30]. Ba/F3 cells stably transduced with gp130 (Ba/F3-gp130) can be activated by Hyper-IL-6 (via gp130/gp130) and IL-27 (WSX-1/gp130), and the cells showed a profound phosphorylation of Akt after stimulation with Hyper-IL-6 or IL-27 (Figure 3B, C). Again, pre-treatment with TBB almost completely blocked the cytokine-induced phosphorylation (Figure 3B, C). Next, we transduced these cells with a cDNA coding for the LIFR (Ba/F3-gp130-LIFR), which renders them responsive towards CT-1, OSM and LIF stimulation (Figure 1A). In line with our previous findings, CT-1 stimulation led to a substantial activation of Akt signaling pathways, which was prevented by CK2 inhibition (Figure 3D).

Activation of the Ras/Raf/MAPK/ERK pathway was also seen after stimulation of Ba/F3 cells with Hyper-IL-6, IL-27 or CT-1 (Figure 3E-G). As expected, inhibition of CK2 prevented ERK phosphorylation. In HepG2 cells, we observed nearly no ERK activation after 15 min of cytokine stimulation, and pERK was only slightly increased after 60 min stimulation with Hyper-IL-6 (Figure 3H). Pre-treatment of HepG2 cells with TBB increased the basal pERK level, but again no strong induction of ERK activity was visible (Figure 3H).

In conclusion, our data highlight an important role for CK2 in the activation of ERK and Akt signaling by IL-6 family cytokines.

### Blockade of CK2 inhibits cytokine-dependent proliferation and STAT activation in Ba/F3-gp130 cells

**Fig 4:**
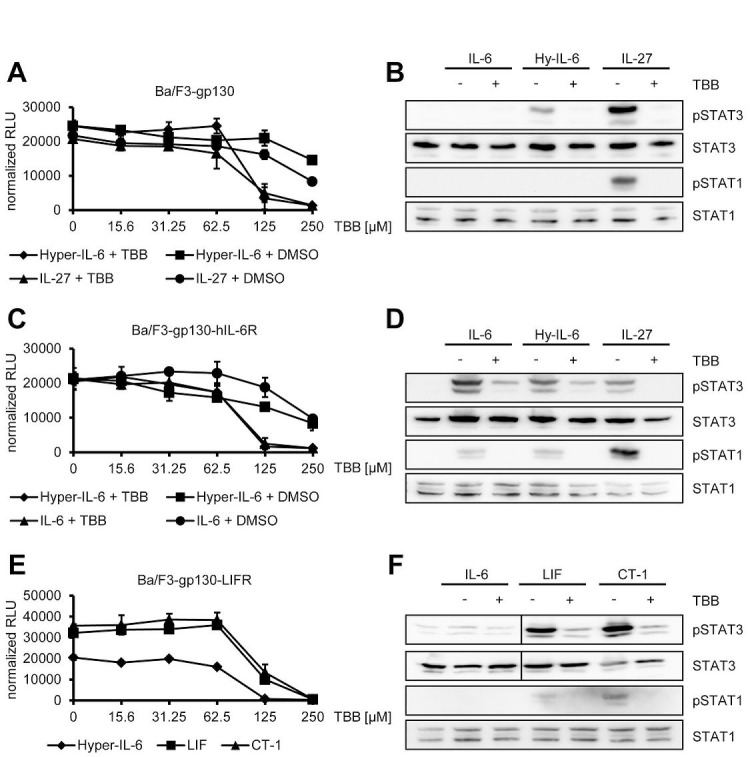
Inhibition of CK2 prevents cytokine-dependent proliferation of Ba/F3-gp130 cells (A) Equal amounts of Ba/F3-gp130 cells were incubated with 10 ng/ml Hyper-IL-6 or 10 ng/ml IL-27 and either increasing amounts of TBB (0-250 μM) or the corresponding amount of DMSO as control. Cellular proliferation was determined as described in Material and Methods. (B) Equal amounts of Ba/F3-gp130 cells were serum-starved for 3 h and either left untreated or where stimulated with 10 ng/ml IL-6, 10 ng/ml Hyper-IL-6 or 10 ng/ml IL-27. Where indicated, cells were pretreated with 100 μM TBB for 90 min prior to cytokine stimulation. Phosphorylation of STAT1 and STAT3 was assessed by Western blotting, and STAT1/STAT3 served as internal loading control, respectively. (C) Equal amounts of Ba/F3-gp130-hIL-6R cells were incubated with 10 ng/ml Hyper-IL-6 or 10 ng/ml IL-6 and either increasing amounts of TBB (0-250 μM) or the corresponding amount of DMSO as control. Cellular proliferation was determined as described in Material and Methods. (D) Equal amounts of Ba/F3-gp130-hIL-6R cells were treated as described in panel (B). Phosphorylation of STAT1 and STAT3 was assessed by Western blotting, and STAT1/STAT3 served as internal loading control, respectively. (E) Equal amounts of Ba/F3-gp130-LIFR cells were incubated with 10 ng/ml Hyper-IL-6, 10 ng/ml LIF or 10 ng/ml CT-1 and increasing amounts of TBB (0-250 μM). Cellular proliferation was determined as described in Material and Methods. (F) Equal amount of Ba/F3-gp130-LIFR cells were treated as described in panel (B). Phosphorylation of STAT1 and STAT3 was assessed by Western blotting, and STAT1/STAT3 served as internal loading control, respectively. Proliferation assays as well as Western Blots show one representative experiment of three performed.

One of the many functions of IL-6 family cytokines is the induction of cellular proliferation. As mentioned above, Ba/F3 cells represent an ideal tool to investigate cytokine-dependent cellular proliferation, since they grow only in the presence of certain cytokines and undergo apoptosis without cytokine stimulation. Thus, Ba/F3-gp130 cells grow in the presence of either Hyper-IL-6 or IL-27, but not IL-6 or without cytokine (Suppl. Figure 1A). To test whether inhibition of CK2 affects cytokine-dependent proliferation of Ba/F3-gp130 cells, we incubated the cells with a constant amount of either Hyper-IL-6 or IL-27 and increasing amounts of TBB (0 – 250 μM). We observed a reduction of cellular proliferation through CK2-blockade towards Hyper-IL-6 (IC_50_: 220 ± 59 μM) and IL-27 (IC_50_: 154 ± 82 μM) in a dose-dependent manner (Figure 4A). As shown before in HepG2 and HeLa cells, TBB blocked the Hyper-IL-6- and IL-27-induced phosphorylation of STAT1 and STAT3 also in Ba/F3-gp130 cells (Figure 4B). Next, we tested the effect of TBB on Ba/F3-gp130-hIL-6R cells, which grow in response to IL-6 stimulation (Suppl. Figure 1B and [31]). Again, TBB decreased cellular proliferation in a dose-dependent manner when cells were stimulated either with Hyper-IL-6 (IC_50_: 119 ± 30 μM) or IL-6 (IC_50_: 187 ± 80 μM) (Figure 4C), and cytokine-dependent phosphorylation of STAT1 and STAT3 was largely absent when cells were pre-treated with TBB (Figure 4D). Finally, we used Ba/F3-gp130 cells stably transduced with LIFR, which made them responsive towards stimulation with LIF and CT-1 (Suppl. Figure 1C). Also in this cell line, TBB decreased cellular proliferation in a dose-dependent manner, irrespective whether the cells were stimulated with Hyper-IL-6 (IC_50_: 79 ± 2 μM), LIF (IC_50_: 147 ± 24 μM) or CT-1 (IC_50_: 162 ± 7 μM) (Figure 4E). As shown in Figure 4F, LIF and CT-1 induced a robust phosphorylation of STAT3 in Ba/F3-gp130-LIFR cells, and this was completely prevented by pre-treatment with TBB. Both cytokines induced only a weak phosphorylation of STAT1, but again this was completely abrogated when CK2 was inhibited (Figure 4F).

We have previously shown that Ba/F3-gp130-hIL-6RΔE317_T352 cells, which lack 36 amino acids within the stalk region of the human IL-6R, proliferate only in response to high concentrations of IL-6 [32]. We therefore asked whether high concentrations of IL-6 family cytokines would be able to induce cellular proliferation even if CK2 is blocked and incubated Ba/F3-gp130 cells with a constant amount of TBB (125 μM) and increasing concentrations of different cytokines (0 – 100 ng/ml). We did not observe a significant increase in cellular proliferation when cells were stimulated with physiological concentrations of IL-6, Hyper-IL-6 or IL-27 (Suppl. Figure 2A-B). However, high concentrations of 100 ng/ml LIF and CT-1 induced weak cellular proliferation (Suppl. Figure 2C).

In conclusion, our data show that pharmacological inhibition of CK2 induces a permanent block of the Jak/STAT signaling pathway that cannot be overcome even at high concentrations of cytokines.

### Activation of CK2 does not induce Jak/STAT activation on its own

**Fig 5:**
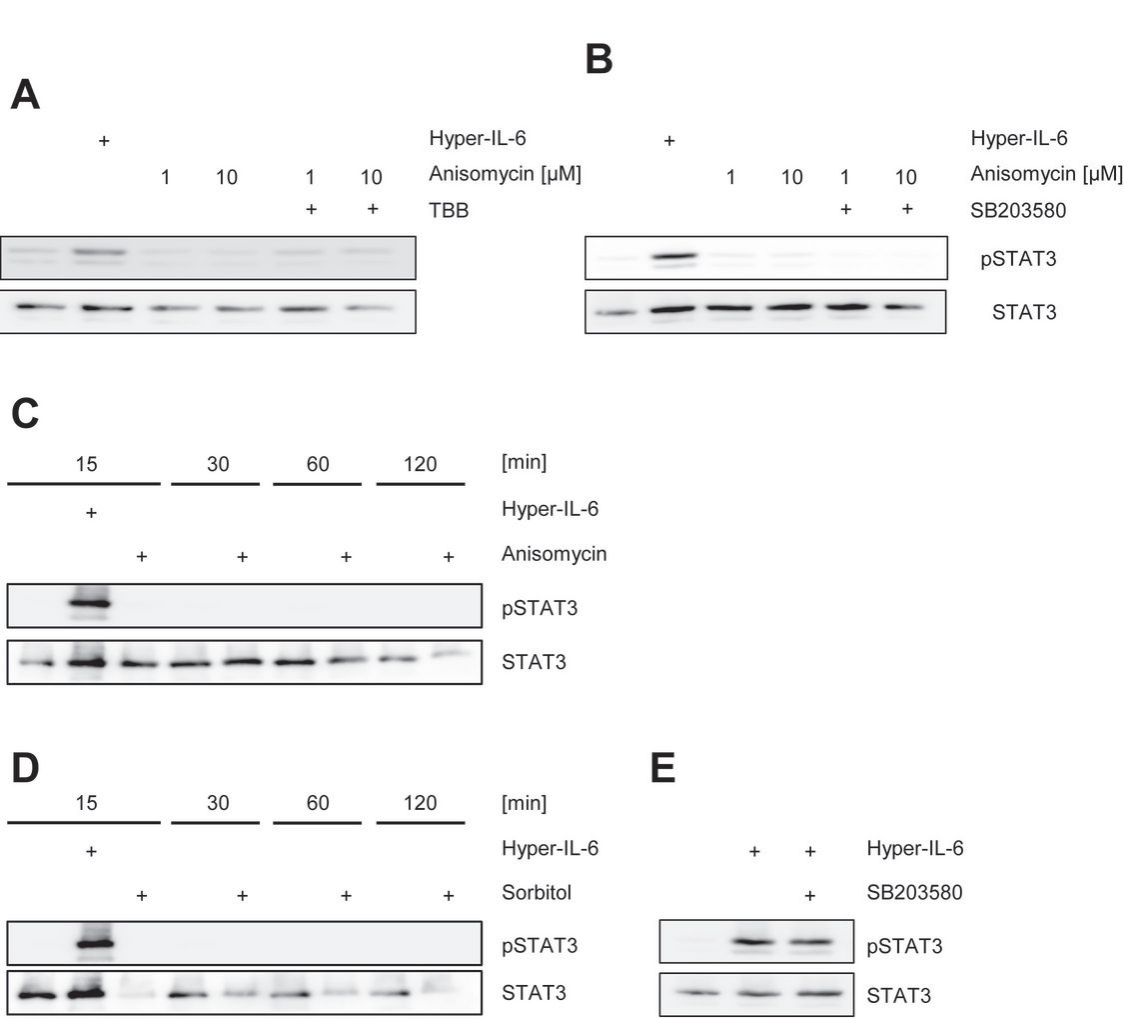
Activation of CK2 does not induce Jak/STAT signaling (A) HepG2 cells were serum starved for 3 h and stimulated with either 10 ng/ml Hyper-IL6 for 15 min, anisomycin for 30 min or left unstimulated. The CK2 inhibitor TBB was added 60 min prior to stimulation where indicated. (B) HepG2 cells were serum-starved for 3 h and stimulated with either 10 ng/ml Hyper-IL6 for 15 min, anisomycin for 30 min or left unstimulated. The p38 MAPK inhibitor SB203580 was added 60 min prior to stimulation where indicated. (C) HepG2 cells were serum-starved for 3 h and either left untreated, were stimulated with 10 ng/ml Hyper-IL-6 or 10 μM anisomycin for 15 min. Furthermore, either untreated or anisomycin-stimulated cells were harvested after 30, 60 or 120 min. (D) HepG2 cells were serum-starved for 3 h and either left untreated, were stimulated with 10 ng/ml Hyper-IL-6 or 500 mM sorbitol for 15 min. Furthermore, either untreated or sorbitol-stimulated cells were harvested after 30, 60 or 120 min. (E) HepG2 cells were serum-starved for 3 h and stimulated with either 10 ng/ml Hyper-IL-6 for 15 min or left unstimulated. The p38 MAPK inhibitor SB203580 was added 60 min prior to stimulation where indicated. Phosphorylation of STAT1 and STAT3 was assessed by Western blotting, and STAT1/STAT3 served as internal loading control, respectively. Shown is one representative Western blot from at least three independent experiments.

Up to this point, our data show that activity of CK2 is necessary for cytokine-induced activation of the Jak/STAT pathway. Next, we asked whether the reciprocal event is possible, i.e. if forced activation of CK2 alone is able to activate Jak/STAT signaling without an extracellular cytokine stimulus. CK2 is constitutively active, but its activity can be enhanced through certain stimuli. The antibiotic anisomycin is known to activate p38 MAPK, which then in turn phosphorylates and activates CK2 [33]. We serum-starved HepG2 cells to reduce the background amount of pSTAT3 and stimulated the cells for 30 min with 1 μM and 10 μM anisomycin. In contrast to stimulation with Hyper-IL-6 for 15 min, we observed no phosphorylation of STAT3 after anisomycin stimulation, and pre-treatment with TBB did not show an effect (Figure 5A). To exclude a role of p38 MAPK in our experimental setup, we incubated HepG2 cells with the p38 MAPK specific inhibitor SB203580, but again did not detect any phosphorylation of STAT3 (Figure 5B).

Activation of the Jak/STAT pathway by cytokines is a rapid mechanism, and usually the first traces of phosphorylated STAT proteins are detected after a few minutes. In contrast, it might be possible that CK2-triggered STAT-phosphorylation is slow and that therefore the 30 min time point in our initial experiments might be inappropriate. We stimulated HepG2 cells with 10 μM anisomycin and monitored pSTAT3 over a time period of 15-120 min, but did not observe any activation (Figure 5C). Next, we repeated the experiment with 500 mM sorbitol, which is also known to activate CK2 [34, 35]. Again, we could not detect phosphorylated STAT3 over a period of up to 2 h (Figure 5D). Since p38 MAPK and CK2 seem to be functionally connected, we asked whether inhibition of p38 MAPK altered Jak/STAT signaling. As shown in Figure 5E, SB203580 did not block Hyper-IL-6-induced phosphorylation of STAT3 in HepG2 cells. We concluded from these experiments that forced activation of CK2 alone was not sufficient to induce Jak/STAT signaling, and that the observed effects solely depended on CK2, but not on p38 MAPK.

### Jak1, but not Jak2, is required for CK2-dependent STAT activation by IL-6 type cytokines

**Fig 6:**
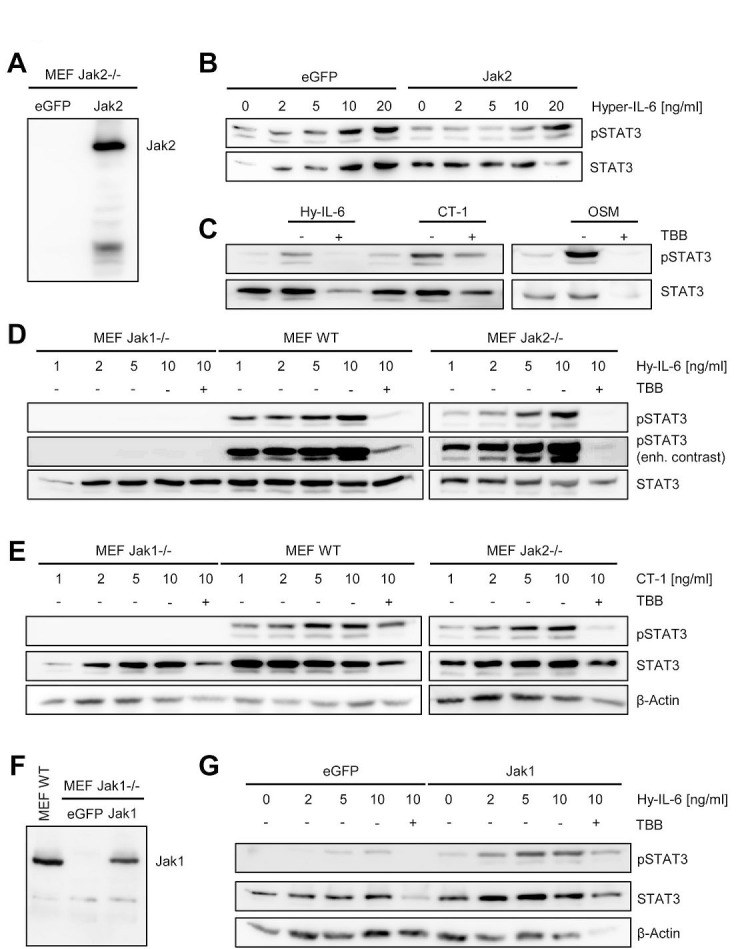
Jak1, but not Jak2, is required for STAT activation by IL-6 family cytokines (A) Jak2^−/−^ MEFs were transiently transfected with a plasmid coding for eGFP or Jak2. Expression of Jak2 was assessed by Western blotting 48 h later. (B) Jak2^−/−^ MEFs transiently transfected with eGFP or Jak2 were stimulated with increasing concentrations of Hyper-IL-6 (0-20 ng/ml) for 15 min. (C) Jak2^−/−^ MEFs transiently transfected with Jak2 were stimulated with 10 ng/ml Hyper-IL-6, CT-1 or OSM for 15 min. Cells were pre-treated with 100 μM TBB for 90 min where indicated. (D, E) Jak1^−/−^, Jak2^−/−^ and wildtype MEFs were stimulated with different concentrations (0-10 ng/ml) of Hyper-IL-6 or CT-1 for 15 min. Cells were pre-treated with 100 μM TBB for 90 min where indicated. (F) Jak1^−/−^ MEFs were transiently transfected with a plasmid coding for eGFP or Jak1. Expression of Jak2 was assessed by Western blotting 48 h later. Lysate of wildtype MEFs served as positive control. (G) Jak1^−/−^ MEFs transiently transfected with eGFP or Jak2 were stimulated with increasing concentrations of Hyper-IL-6 (0-20 ng/ml) for 15 min. Cells were pre-treated with 100 μM TBB for 90 min where indicated. Phosphorylation of STAT3 was determined in all experiments by Western blotting, and STAT3 as well as β-actin served as loading controls. Shown is one representative Western blot from at least three independent experiments.

Several kinases are able to phosphorylate STAT1 and STAT3 proteins. Cytokines which signal through gp130 can activate Jak1, Jak2 and Tyk2, which subsequently phosphorylate certain STATs [2]. To address the involvement of different kinases in CK2-dependent STAT activation, we made use of kinase-deficient murine embryonic fibroblasts (MEFs). Zheng et al. suggested that blockade of CK2 impairs the ability of Jak2 to phosphorylate STAT1 and STAT3 [14]. This seemed rather unlikely, as Jak1 has been shown to be the dominant kinase in gp130-mediated signaling [36]. Jak2^−/−^ MEFs express no detectable Jak2 protein, but can be reconstituted via overexpression of a Jak2 coding cDNA (Figure 6A). After stimulation with Hyper-IL-6, both Jak2^−/−^ and Jak2-reconstituted Jak2^−/−^ MEFs induced STAT3 phosphorylation (Figure 6B). Furthermore, stimulation with CT-1 and OSM induced STAT3 phosphorylation in the absence of Jak2, which was inhibited by blockade of CK2 with TBB (Figure 6C). These results suggest that Jak2 is not the main kinase activated after stimulation with Hyper-IL-6, CT-1 or OSM, questioning the proposed mechanism by Zheng et al. [14]. Therefore, we repeated these experiments with Jak1^−/−^ MEFs. As shown in Figure 6D, phosphorylated STAT3 was detected in a concentration-dependent manner when we stimulated wildtype or Jak2^−/−^ MEFs. A small portion of phosphorylated STAT3 appeared in Jak1^−/−^ MEFs only with strongly enhanced contrast (Figure 6D, lower panel). We obtained similar results with CT-1 (Figure 6E). Importantly, in Jak1^−/−^ MEFs reconstituted with Jak1 (Figure 6F), we observed STAT3 phosphorylation after treatment with Hyper-IL-6, and this could be blocked by inhibition of CK2 (Figure 6G). In conclusion, we showed that Jak1, rather than Jak2 is the kinase that is affected by inhibition of CK2 upon IL-6 type cytokine stimulation. Our data did, however, not exclude a functional role of CK2 in Jak2 activation.

### Signaling of a constitutively active gp130 variant is blocked by CK2 inhibition

**Fig 7:**
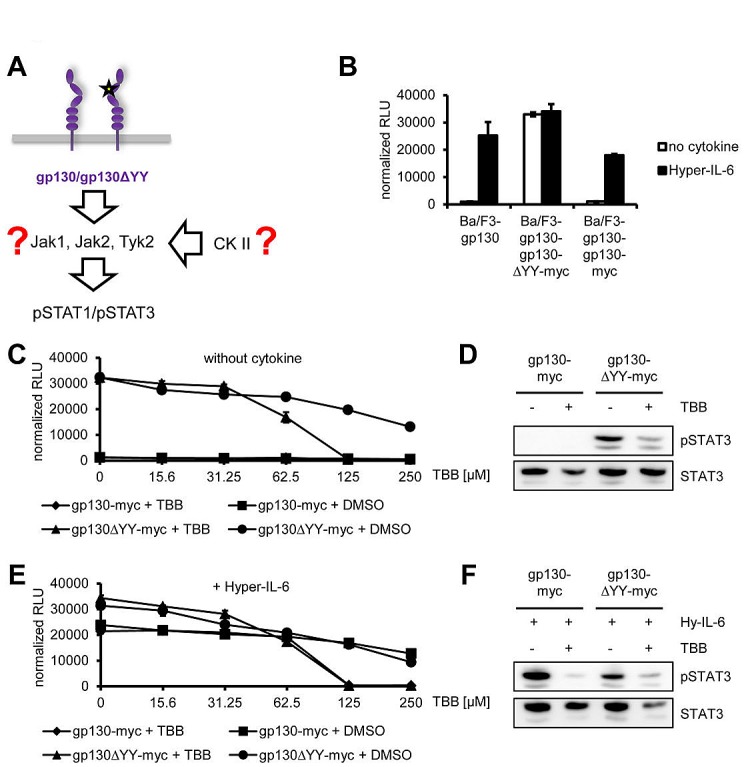
Jak1-dependent STAT-activation of the constitutively active gp130ΔYY-mutant depends on CK2 (A) Schematic representation of the gp130ΔYY-mutant harboring a deletion in domain 2. The kinase leading to constitutive STAT activation and the role of CK2 in this are unknown. (B) Equal amounts of Ba/F3-gp130, Ba/F3-gp130-gp130-myc and Ba/F3-gp130-gp130ΔYY-myc cells were incubated with or without 10 ng/ml Hyper-IL-6. (C) Equal amounts of Ba/F3-gp130-gp130-myc and Ba/F3-gp130-gp130ΔYY-myc cells were incubated with either increasing amounts of TBB (0-250 μM) or the corresponding amount of DMSO as control without any cytokine. (D) Equal amounts of Ba/F3-gp130-gp130-myc and Ba/F3-gp130-gp130ΔYY-myc cells were serum-starved for 3 h, and cells were pretreated with 100 μM TBB for 90 min prior to cytokine stimulation where indicated. Phosphorylation of STAT1 and STAT3 was assessed by Western blotting, and STAT1/STAT3 served as internal loading control, respectively. (E) Cells were treated as described under panel (B), but 10 ng/ml Hyper-IL-6 was added. (F) Cells were treated as described under panel (D), but cells were stimulated with 10 ng/ml Hyper-IL-6 for 15min. Phosphorylation of STAT1 and STAT3 was assessed by Western blotting, and STAT1/STAT3 served as internal loading control, respectively. Cellular proliferation in all assays shown was determined as described in Material and Methods. Proliferation assays as well as Western Blots are one representative experiment of three performed.

Next, we asked whether cytokine-independent activation of the Jak/STAT signaling pathway can also be blocked by CK2 inhibition. Many inflammatory hepatocellular adenomas (IHCAs) have been described to be often characterized by gain-of-function mutations within gp130, which led to ligand-independent constitutive activation [37]. Introduction of one of these mutants, which harbors a deletion of Tyr-186 to Tyr-190 (gp130ΔYY), into Ba/F3-gp130 cells led to cytokine-independent phosphorylation of STAT3 and cellular proliferation, which was blocked by an anti-gp130 antibody which specifically neutralizes IL-11 signaling in wildtype gp130 [38]. The responsible kinase was recently identified as Jak1 [39], but it is unknown whether CK2 is needed for the constitutive gp130 activation (Figure 7A).

As shown before [38, 40, 41], Ba/F3-gp130 cells stably transduced with gp130ΔYY grew cytokine-independently, whereas the parental Ba/F3-gp130 cell line or Ba/F3-gp130 cells stably transduced for a second time with wild-type gp130 only proliferated in the presence of Hyper-IL-6 (Figure 7B). Next, we incubated Ba/F3-gp130-gp130ΔYY-myc cells with increasing amounts of TBB (0 – 250 μM) without any cytokine (Figure 7C) and observed a TBB-concentration-dependent inhibition of cellular proliferation (IC_50_: 70 ± 15 μM). As expected, the control cell line Ba/F3-gp130-gp130-myc showed no proliferation without cytokine stimulation (Figure 7C). In line with this, TBB was able to reduce the amount of constitutively phosphorylated STAT1 and STAT3 in Ba/F3-gp130-gp130ΔYY-myc cells (Figure 7D). We performed a similar experiment with an additional stimulation by Hyper-IL-6, but observed again a TBB-concentration-dependent inhibition of cellular proliferation of Ba/F3-gp130-gp130ΔYY-myc (IC_50_: 65 ± 6 μM) and Ba/F3-gp130-gp130-myc (IC_50_: 150 ± 23 μM) cells (Figure 7E). This was accompanied by reduction of pSTAT1 and pSTAT3 in both cell lines upon TBB treatment (Figure 7F). Thus, our results conclusively showed that TBB suppressed Jak1-dependent signaling of a constitutively active gp130 variant, and that this inhibition could not be overcome by the addition of exogenous Hyper-IL-6.

### The constitutively active STAT3 variant STAT3Y640F needs CK2 activity for signaling

Recently, somatic STAT3 mutations have been found in human inflammatory hepatocellular adenomas [42] and large granular lymphocytic leukemia [43]. These mutations resulted in an increase in hydrophobicity within the Src homology 2 (SH2) domain of STAT3, which is required for STAT3 dimerization. As a consequence, the mutations led to increased STAT3 phosphorylation in the absence of any stimulus.

**Fig 8:**
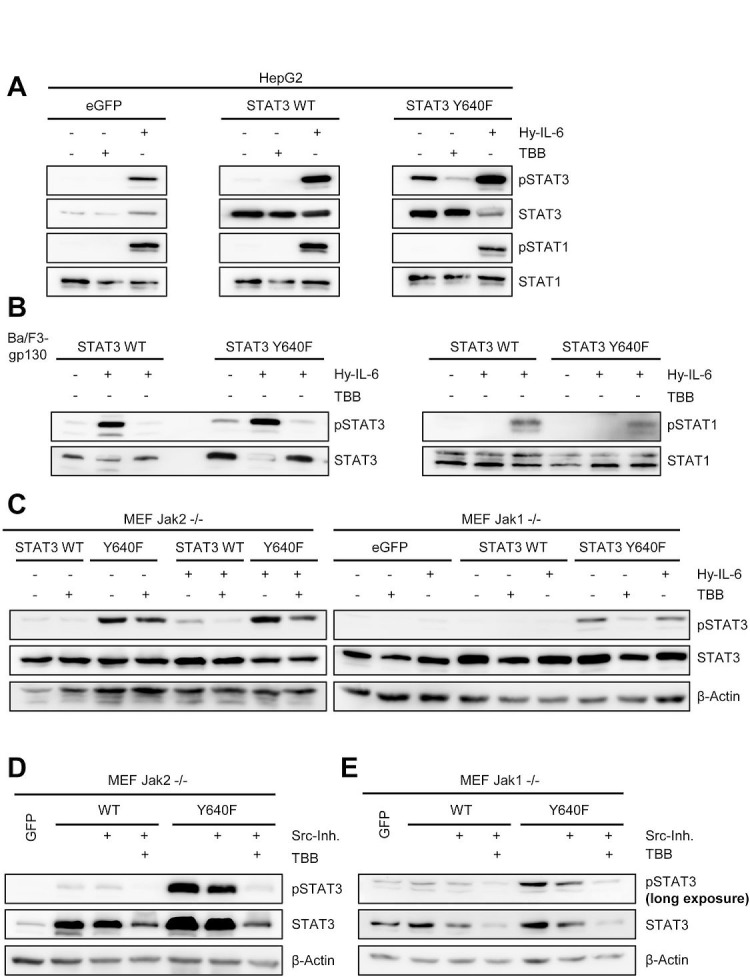
Activity of CK2 is required for phosphorylation of a constitutively active STAT3 mutant (A) HepG2 cells were transiently transfected with eGFP, STAT3wt or STAT3Y640F. Two days later, cells were serum-starved for 5h and stimulated with either 10 ng/ml Hyper-IL-6 or were pre-treated with 100 μM TBB for 90min where indicated. (B) Ba/F3-gp130 cells stably transduced with STAT3wt or STAT3Y640F were serum-starved for 3 h and stimulated with either 10 ng/ml Hyper-IL-6 or were pre-treated with 100 μM TBB for 90 min where indicated. Phosphorylation of STAT1 and STAT3 was determined by Western blotting, and STAT1/3 served as internal loading control. (C) Jak2^−/−^ or Jak1^−/−^ MEFs were transiently transfected with a plasmid coding for eGFP, STAT3wt or STAT3Y640F. Two days later, cells were serum-starved for 5 h and stimulated with either 10 ng/ml Hyper-IL-6 or were pre-treated with 75 μM TBB for 90 min where indicated. Phosphorylation of STAT3 was determined by Western blotting, and STAT3/β-actin served as internal loading control. (D, E) Jak2^−/−^ or Jak1^−/−^ MEFs were transiently transfected with a plasmid coding for eGFP, STAT3wt or STAT3Y640F. Two days later, cells were serum-starved for 5 h and treated with Src-inhibitor alone or Src-inhibitor in combination with TBB for 90 min. Phosphorylation of STAT3 was determined by Western blotting, and STAT3/β-actin served as internal loading control. Western Blots show one representative experiment of three performed.

We asked whether phosphorylation of the constitutive STAT3 variant Y640F was also dependent on CK2 activity and therefore introduced the Y640F mutation into STAT3, which is the most frequently found mutation in STAT3 [43]. First, we transfected HepG2 cells with either eGFP, wildtype STAT3 (STAT3wt) or STAT3 with the Y640F mutation (STAT3Y640F) (Figure 8A). No constitutive STAT3 phosphorylation was observed in eGFP or STAT3wt transfected cells. In contrast, HepG2 cells transfected with STAT3Y640F displayed constitutive STAT3 phosphorylation. Importantly, no STAT1 phosphorylation was detectable in all three cell lines. We could significantly reduce pSTAT3 in STAT3Y640F transfected cells by TBB (Figure 8A), which strongly suggests an involvement of CK2 in the constitutive phosphorylation of STAT3Y640F. As a control, Hyper-IL-6 induced pSTAT1 and pSTAT3 in all three cell lines.

To confirm our results obtained in HepG2 cells, we generated stably transduced Ba/F3-gp130 cells with either wildtype STAT3 or STAT3Y640F. In line with our previous findings, overexpression of STAT3Y640F led to constitutive STAT3 phosphorylation, which was blocked by TBB-induced CK2-inhibition. Again, we observed no STAT1 phosphorylation when STAT3Y640F was overexpressed, and no constitutive STAT activation in the STAT3wt expressing cells (Figure 8B).

To identify the kinase that activates and phosphorylates STAT3Y640F, we transiently transfected Jak2^−/−^ and Jak1^−/−^ MEFs with STAT3wt and STAT3Y640F (Figure 8C). STAT3Y640F was constitutively phosphorylated in Jak2^−/−^ cells, showing again that Jak2 was not among the responsible kinases. Interestingly, STAT3Y640F was also phosphorylated in Jak1^−/−^ MEFs, although to a lesser degree than in Jak2^−/−^ MEFs. However, transfection of the different STAT3 variants into Jak1^−/−^ MEFs was less effective, hampering a direct comparison. Nevertheless, a previous report by Pilati et al. showed that Jak1 contributes to the constitutive phosphorylation of STAT3Y640F [42]. Furthermore, the authors suggested the involvement of Src kinase. To address this, we treated STAT3wt- and STAT3Y640F-transfected Jak1^−/−^ and Jak2^−/−^ MEFs with the Src kinase inhibitor 1, which reduced constitutive phosphorylation of STAT3Y640F in both MEFs (Figure 8D, E). It should be noted, however, that in Jak2^−/−^ MEFs STAT3Y640F phosphorylation was still detectable when Src was inhibited, underlining the major contribution of Jak1. In Jak1^−/−^ MEFs, the already low constitutive phosphorylation of STAT3Y640F was further reduced by Src kinase inhibition (Figure 8E). Most strikingly, when we combined Src kinase and CK2 inhibitors, the phosphorylation of STAT3Y640F was completely reduced to baseline levels.

In summary, our data indicate that although Jak1 and Src kinase are involved in the phosphorylation of STAT3Y640F, also here CK2 plays a pivotal role. It is tempting to speculate that either CK2 activates an additional kinase that in turn phosphorylates STAT3Y640F, or that STAT3Y640F is a direct substrate of CK2.

## DISCUSSION

Cytokines of the IL-6 family are profound activators of intracellular signaling pathways, notably the Jak/STAT pathway. This signaling cascade is tightly controlled through several negative feedback loops, and dysregulated Jak/STAT signaling is associated with inflammation and cancer [6, 44].

Among the seven members of the STAT family, the phosphorylation of STAT1 and STAT3 seems to be of major importance with regard to IL-6 type cytokines [3]. Activation of STAT1 and STAT3 leads to target gene transcription, and thus is a key driver of cellular proliferation. Binding of IL-6 family cytokines to their specific receptor complexes on the cell surface leads to β-receptor homo- or heterodimerization and subsequently activation of specific kinases, namely Jak1, Jak2 and Tyk2, which are constitutively associated with gp130, LIFR, OSMR and WSX-1. Receptor dimerization results in auto-/transphosphorylaton of the Jak kinases and of specific tyrosine residues within the intracellular domains of the β-receptors, which in turn serve as docking sites for STAT family proteins. Subsequently, JAKs phosphorylate STATs, resulting in STAT-dissociation from the receptor chain, formation of homo- and heterodimers, and translocation into the nucleus where they act as transcription factors. Thus, several inhibitors of Jak family members have been developed and are currently used therapeutically [2, 45].

**Fig 9:**
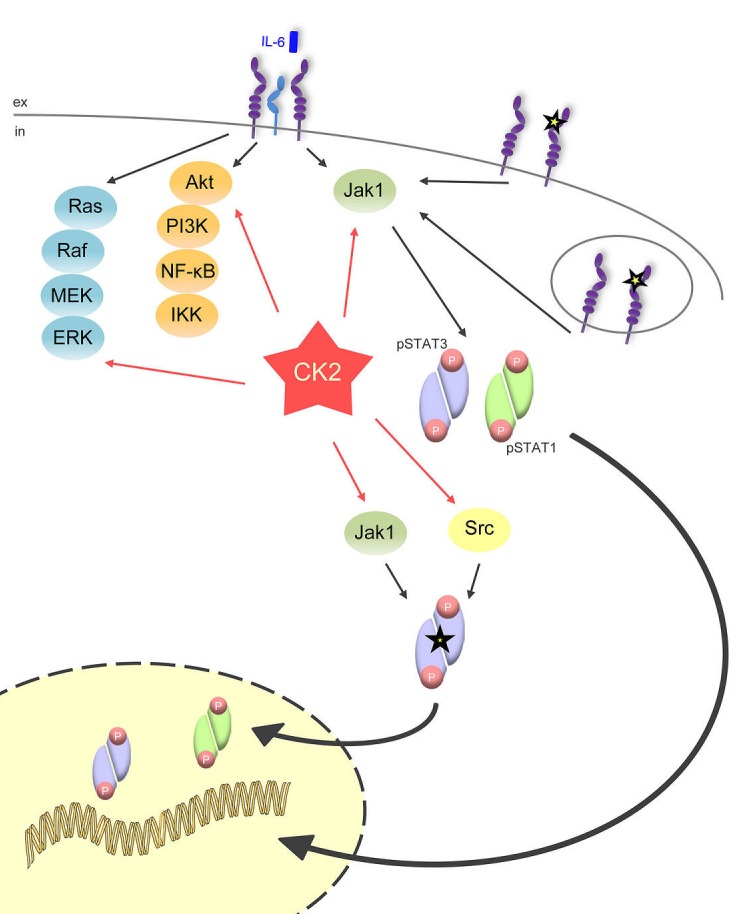
CK2 is the central lynchpin of the signaling pathways investigated in this study The schematic overview shows that IL-6 family cytokines activate the three major signaling pathways Jak/STAT, Ras/Raf/MEK/ERK, and Akt/PI3K. All of them need CK2 activity for the initiation of downstream signaling. The gp130ΔYY-mutant, which is constitutively active and signals from the cell membrane and from intracellular compartments, solely activates Jak/STAT signaling, and this can be efficiently blocked through CK2 blockade. A constitutively active STAT3 mutant (STAT3Y640F), which is constitutively phosphorylated by Jak1 and Src kinase, also needs CK2 activity

In the current study, we add another player to the complex intracellular network that controls proper Jak/STAT signaling (Figure 9). Inhibition of CK2 efficiently prevented the phosphorylation of STAT1 and STAT3 induced by all members of the IL-6 family of cytokines. This shows that the activity of CK2 is a prerequisite to allow activation of the Jak/STAT signaling pathway, irrespective of the cytokine stimulus. Accordingly, blockade of CK2 inhibited the proliferation of different Ba/F3-gp130 cell lines, which depend upon appropriate cytokine stimulation for proliferation.

Three different members of the Jak family (Jak1, Jak2 and Tyk2) have been implicated in IL-6 type cytokine signal transduction [46]. Recently, Zheng et al. have proposed a CK2-dependent role for Jak2 in OSM-mediated signaling [14], and they demonstrated a physical interaction between CK2 and Jak2 as well as Jak1. Furthermore, autophosphorylation of JAK2V617F, a constitutive active Jak2 variant found in myeloproliferative diseases, was inhibited after CK2 suppression [14]. The authors predicted several CK2 dependent phosphorylation sites in Jak2, which, however, were not characterized on molecular and functional level thus far.

The activity of Jak2 is, however, dispensable for signal transduction by LIF, IL-6 and other IL-6 family cytokines ([47] and this study). In line with these previous findings, we detected unaltered STAT3 phosphorylation in MEFs deficient for Jak2. Importantly, IL-6 type cytokine-induced STAT3 phosphorylation was dependent on CK2 in Jak2-deficient MEFs. In sharp contrast, Jak1 deficiency resulted in a nearly complete loss of STAT3 activation when cells were treated with Hyper-IL-6 or CT-1, arguing that Jak1, but not Jak2, is the critical kinase which needs CK2 activity to phosphorylate its substrates.

Besides induced activation of STAT3 by IL-6 type cytokines, STAT3 can be constitutively activated through permanently dimerized gp130. First described in an artificial cell system [48], small somatic deletions in gp130 that lead to constitutive dimerization have later been shown to be involved in the development of human inflammatory hepatocellular adenomas [37]. The constitutive activation of STAT3 was blocked with an anti-gp130 antibody that selectively inhibits IL-11 signaling [38], or a small chemical inhibitor of Jak kinases [41]. Recently, it was shown that this activation of STAT3 was dependent upon Jak1, but not Jak2 or Tyk2 activity [39], which supported our conclusions. Interestingly, STAT3 phosphorylation and cellular proliferation of Ba/F3 cells induced by the ligand-independent gp130YY variant was also blocked after inhibition of CK2, suggesting that inhibition of CK2 might be a therapeutic option for the treatment of constitutive-active gp130-dependent hepatic adenomas.

Recently, mutations of STAT3 have been described that lead to the development of human inflammatory hepatocellular adenomas [42] or large granular lymphotic leukemia [43]. The mutation STAT3Y640F, which was observed most frequently, confers constitutive STAT3 phosphorylation. Tyrosine phosphorylation of the constitutive active STAT3Y640F variant was also detected in Jak1 and Jak2 deficient MEFs. CK2 inhibition almost completely blocked STAT3Y640F phosphorylation in Jak1 deficient MEFs and to a lesser extent in Jak2 deficient MEFs. It was recently described that Src kinase contributes to phosphorylation of STAT3Y640F [42]. Whereas Scr inhibition partly inhibited phosphorylation of STAT3Y640F, co-inhibition of Src and CK2 completely blocked phosphorylation of STAT3Y640F. Currently, it is unclear whether CK2 activates an additional kinase that in turn phosphorylates STAT3Y640F, or if STAT3Y640F is a direct substrate of CK2. Irrespective of this, CK2 blockade might be a valuable therapeutic option in the treatment of STAT3Y640F-driven leukemia.

Constitutively active gp130 mutants and STAT3Y640F are examples of mutations in the gp130/Jak/STAT signaling cascade which are directly associated with human cancer development. In addition, expression of IL-6 family cytokines is upregulated in many human cancers. For example, IL-6 was increased in patients suffering from colitis-associated cancer (CAC) [49], and autocrine IL-6 signaling was shown to be critically involved in lung and breast cancer development [50, 51]. Thus, inhibition of IL-6/gp130 signaling is considered as a valuable therapeutic strategy [52]. Increased expression of IL-11 was found in biopsies from human gastric cancer patients [53], and IL-11 has been shown to be the critical IL-6 family cytokine in gastric tumor formation [54]. Therapeutic targeting of IL-11 was also beneficial in gastrointestinal tumorigenesis [55]. These examples demonstrate that increased IL-6 family signaling is highly associated with tumor initiation and progression.

The ability of dimerized STAT3 to transform cells has led to the assumption that STAT3 can be considered an oncogene [56]. The activity and expression of CK2 is increased in many human tumors [20]. Our data show a close connection between the activity of CK2 and the constitutive and induced activation of the Jak/STAT signaling pathway. Several chemical compounds, which act as ATP-competitive inhibitors, have been shown to selectively target CK2. One of them, CX-4945, has recently completed phase I clinical studies with promising results for the treatment of different solid tumors [57].

In conclusion, our data highlight an important role of CK2 in Jak/STAT signaling and favor the inhibition of CK2 as a valid therapeutic option in STAT3-driven malignancies (Figure 9).

## MATERIALS AND METHODS

### Cells and Reagents

The parental Ba/F3-gp130 cells and retroviral transduced Ba/F3-gp130-hIL-6R and Ba/F3-gp130-LIFR cells have been described previously [58, 59]. HepG2 and HeLa cells were obtained from DMSZ (Braunschweig, Germany). All cells used in this study were grown in DMEM high glucose culture medium (Gibco, Life Technologies, Grand Island, NY, USA) supplemented with 10% fetal bovine serum, penicillin (60 mg/l) and streptomycin (100 mg/l) at 37°C in an incubator with 5% CO_2_ in a water-saturated atmosphere. Ba/F3-gp130 cells were cultured using 10 ng/ml recombinant Hyper-IL-6 (fusion protein of IL-6 and the sIL-6R connected by a peptide linker), which was expressed and purified as described previously [25, 60]. After stable transduction with hIL-6R, cells were cultured with 10 ng/ml recombinant human IL-6. Human IL-6 was expressed and purified as described previously [61]. Recombinant IL-11 and OSM were purchased from ImmunoTools (Friesoythe, Germany). IL-27 and CT-1 were from R&D Systems (Minneapolis, MN, USA). Emodin, TBB, anisomycin, sorbitol, 4-(4′-Phenoxyanilino)-6,7-dimethoxyquinazoline,6,7-Dimethoxy-N-(4-phenoxyphenyl)-4-quinazolinamine (Src Inhibitor-1) and SB203580 were purchased from Sigma-Aldrich (Steinheim, Germany). Plasmids coding for the gp130 mutant ΔYY and stable transduced Ba/F3-gp130 cells thereof have been described previously [38]. Expression plasmids for murine Jak1 and Jak2 have been described previously [62]. Jak1^−/−^ MEFs have been described previously [47]. The anti-phospho STAT1 (Y701) (58D6), anti-STAT1, anti-phospho STAT3 (Y705) (D3A7), anti-STAT3 (124H6), anti-Jak1 (6G4), anti-Jak2 (D2E12), anti-phospho-ERK1/2 (T202/Y204), anti ERK, anti-phospho-Akt (S473) (D9E) and anti-Akt mAbs were purchased from Cell Signaling Technology. Anti-β-Actin (C4) mAb was obtained from Santa Cruz.

### LIF production

Human leukemia inhibitory factor (LIF) was expressed in pGEX2T vector as a glutathione S-transferase (GST) fusion protein as described previously [63] with minor changes in BL21(DE3) *E. coli* cells. Briefly, freshly transformed cells were grown to an O.D._600_ of 0.5 and induced by 400 μM IPTG at 30°C for 12 h. Cells were harvested, sonicated, and treated with DNaseI (7.5 μg/ml) and lysozyme (75 μg/ml). The LIF-GST fusion protein was captured with glutathione-Sepharose 4B beads (GE Healthcare, Munich, Germany) and the LIF moiety was released by thrombin cleavage (GE Healthcare). Purity of sterile filtered LIF was checked via Coomassie stained SDS-PAGE, concentration was determined with Bradford assay, and biological activity was tested using Ba/F3-gp130-LIFR cells and mouse ES cell line EB5 [63, 64]. A detailed protocol is available on request.

### Plasmid construction

The cDNA for STAT3 was excised from pcEP4 with KpnI and EcoRV and the sticky ends were blunted using Klenow fragment (Thermo Scientific). The resulting DNA fragment was subcloned into pCR-Script vector. Mutagenesis for the generation of STAT3 Y640F was performed using standard techniques with the following primers: 1Fwd: AGAAGCTCCT AGGGCCTGGT, 2Rev: TGCTTGGTGAATGGCTCTAC, 3Fwd: GTAGAGCCATTCACCAAGCA, 4Rev: TATCATGTCTGGATCCCCCA. The PCR product was cloned into pCR-Script using AvrII and BamHI and subcloned into the expression vector pcEP4 applying NotI and BamHI.

### Transient transfection of HepG2 cells and murine embryonic fibroblasts (MEFs)

For transient transfection cells were seeded on 10 cm dishes at a density of 6x10^5^ (HepG2) or 7x10^5^ (MEFs) the day before. TurboFect (Thermo Scientific) or JetPrime (Polyplus) were used as transfection reagents according to the manufacturer's instructions.

### Cytokine stimulation of HepG2, HeLa, MEF and Ba/F3-gp130 cells

HepG2 and HeLa cells were seeded on 6-well-plates at a density of 7.5x10^5^ cells/well and 2.5x10^5^ cells/well respectively. For stimulation of MEFs 4x10^5^ cells were seeded per well. After 16 h, cells were washed and starved in serum-free medium for 5 h. In case of CK2-inhibition TBB was added at a concentration of 100 μM (or 75 μM for MEFs) 90 min prior to cytokine stimulation. When not indicated, the concentration of cytokines was 10 ng/ml and cells were stimulated for 15 min at 37°C.

### Cell lysis and Western blotting

After stimulation cells were scraped in 1 ml PBS on ice. The pellet was lysed in 75 μl or 250 μl (6-well or 10 cm dish respectively) lysis buffer containing 50 mM Tris, pH 7.5, 150 mM NaCl, 2 mM EDTA, 1 mM NaF, 1 mM Na_3_VO_4_, 1% IGEPAL [NP-40], 1% Triton X-100 and complete protease inhibitor cocktail tablets (Roche, Grenzach, Germany). The concentration of total protein in the lysates was determined using BCA Protein Assay kit (Thermo Scientific) following the manufacturer's instructions. 50 μg protein were loaded onto an 8 or 10% SDS-Gel and subsequently blotted onto a PVDF membrane. The membranes were then blocked with 5% skim milk powder in TBS-T (10 mM Tris-HCl, pH 7.6, 150 mM NaCl, and 0.05% Tween 20) for 1 h at room temperature. Primary antibodies were applied overnight at 4°C in 5% skim milk powder or 5% BSA in TBS-T.

Between incubation with two different primary antibodies membranes were incubated with stripping buffer (62.5 mM Tris-HCl, pH 6.8, 2% SDS, 0.1% β-mercaptoethanol) for 30 min at 65°C and subsequently washed in TBS-T and blocked again. The detection of proteins was performed using ECL prime reagent (GE Healthcare).

### Retroviral transduction of murine Ba/F3-gp130 cells

Retroviral transduction using Phoenix Eco cell supernatant has been described elsewhere [65, 66]. Here, STAT3wt and STAT3Y640F were subcloned into the retroviral pMOWS vector, Phoenix cells were transiently transfected, supernatants collected, and Ba/F3-gp130 cells transduced. Cells were selected afterwards for puromycin resistance and cultivated in the presence of 10 ng/ml Hyper-IL-6 throughout the transduction.

### Proliferation assays

The Cell Titer Blue Cell viability assay reagent (Promega, Karlsruhe, Germany) was applied as described previously [7, 31] to determine proliferation of the different Ba/F3-gp130 cell lines according to the manufacturer's protocol. To measure the extinction, a Tecan infinite M200 PRO reader (excitation 560 nm, emission 590 nm, gain 90, i-control 1.7 software, Tecan AG, Maennedorf, Switzerland) was used. Normalization of relative light units (RLU) was achieved by subtraction of negative control values. All values were measured in triplicates per experiment.

### Calculation of IC_50_ values

The IC_50_ values, indicating the amount of inhibitor necessary to block half of the cellular proliferation, were calculated with GraphPad Prism 5 (GraphPad Software Inc., La Jolla, CA, USA).

## SUPPLEMENTARY FIGURES


