# Supplementary material for: Inhibition of protein kinase II (CK2) prevents induced signal transducer and activator of transcription (STAT) 1/3 and constitutive STAT3 activation
Source: Oncotarget. 2014 Mar 23;5(8):2131–48. doi: 10.18632/oncotarget.1852 (PMC4039151; doi:10.18632/oncotarget.1852)
Supplement: Supplementary file 1 [file oncotarget-05-2131-s001.pdf]

Inhibition of Protein Kinase II (CK2) prevents induced Signal Transducer and Activator of Transcription (STAT) 1/3 and constitutive STAT3 activation - Aparicio-Siegmund et al

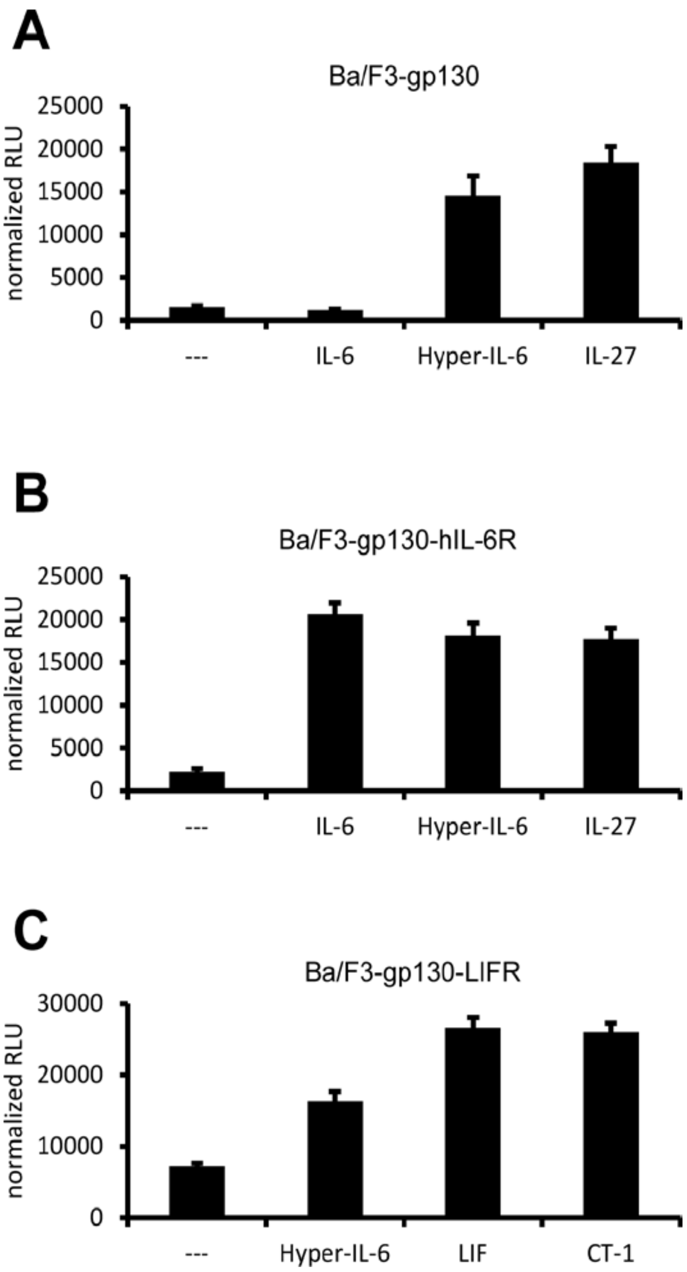

Suppl. Fig. 1: Cytokine-dependent proliferation of different Ba/F3-gp130 cell lines. (A-C) Equal amounts of (A) Ba/F3-gp130, (B) Ba/F3-gp130-hIL-6R and (C) Ba/F3-gp130-LIFR cells were

incubated with 10 ng/ml of the indicated cytokines. Cellular proliferation in all assays shown was determined as described in Material and Methods.

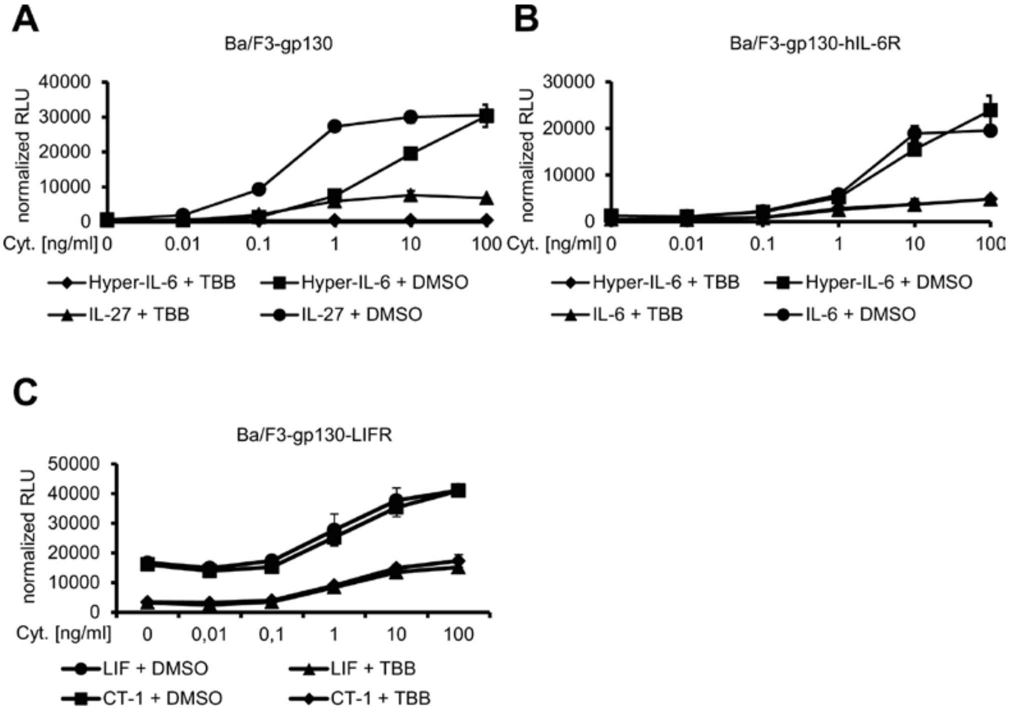

Suppl. Fig. 2: High cytokine concentrations do not overcome CK2 blockade. (A) Equal amounts of Ba/F3-gp130 cells were incubated with 125 µM TBB or the appropriate amount of DMSO as control and increasing amounts of either Hyper-IL-6 (0-100 ng/ml) or IL-27 (0-100 ng/ml). (B) Equal amounts of Ba/F3-gp130-hIL-6R cells were incubated with 125 µM TBB or the appropriate amount of DMSO as control and increasing amounts of either Hyper-IL-6 (0-100 ng/ml) or IL-6 (0-100 ng/ml). (C) Equal amounts of Ba/F3-gp130-LIFR cells were incubated with 125 µM TBB or the appropriate amount of DMSO as control and increasing amounts of either LIF (0-100 ng/ml) or CT-1 (0-100 ng/ml). Cellular proliferation was determined as described in Material and Methods. The data shown are one representative experiment of three performed.
